# Development of a Real-Time Cell Analysing (RTCA) method as a fast and accurate screen for the selection of chikungunya virus replication inhibitors

**DOI:** 10.1186/s13071-015-1104-y

**Published:** 2015-11-09

**Authors:** Suria Marlina, Meng-Hooi Shu, Sazaly AbuBakar, Keivan Zandi

**Affiliations:** Tropical Infectious Diseases Research and Education Centre (TIDREC), Department of Medical Microbiology, Faculty of Medicine, University of Malaya, Kuala Lumpur, Malaysia

**Keywords:** Real-time cell analysis, Cytopathogenicity, Antiviral screening, Chikungunya, Ribavirin

## Abstract

**Background:**

The xCELLigence real-time cell analysis (RTCA) system is an established electronic cell sensor array. This system uses microelectronic biosensor technology that is verified for real-time, label-free, dynamic and non-offensive monitoring of cellular features, including detection of viral cytopathic effect (CPE). Screening viral replication inhibitors based on presence of CPE has been applied for different viruses, including chikungunya virus (CHIKV). However, most CPE-based methods, including MTT and MTS assays, do not provide information on the initiation of CPE nor the changes in reaction rate of the virus propagation over time. Therefore, in this study we developed an RTCA method as an accurate and time-based screen for antiviral compounds against CHIKV.

**Methods:**

CHIKV-infected Vero cells were used as an *in vitro* model to establish the suitability of the RTCA system as a quantitative analysis method based on the induction of CPE. We also performed an MTS assay as a CPE-based conventional method. Experimental assays were carried out to evaluate the optimal seeding density of the Vero cells, cytotoxicity of the tested compounds, titration of CHIKV and the antiviral activity of ribavirin, which has been reported as an effective compound against CHIKV *in vitro* replication.

**Results:**

The optimal time point for viral inoculation was 18 h after seeding the cells. We determined that the maximum non-toxic dose (MNTD) of ribavirin was 200 μg/ml for Vero cells. Regarding the dynamic monitoring of Vero cell properties during antiviral assay, approximately 34 h post-infection, the normalised Cell Index (CI) values of CHIKV-infected Vero cells started to decrease, while the vehicle controls did not show any significant changes. We also successfully showed the dose dependent manner of ribavirin as an approved *in vitro* inhibitor for CHIKV through our RTCA experiment.

**Conclusion:**

RTCA technology could become the prevailing tool in antiviral research due to its accurate output and the opportunity to carry out quality control and technical optimisation.

## Background

Chikungunya virus (CHIKV) is an enveloped arbovirus with a positive-sense single-stranded RNA genome and belongs to the *Togaviridae* family [1]. CHIKV is transmitted to humans via bites from infected *Aedes* mosquitoes. CHIKV can be detected as early as 4 days post-infection in the saliva of the mosquitoes, which indicates a short period of extrinsic incubation [2]. Chikungunya is a Makonde word for “that which bends up” describing the contorted posture and unbearably painful rheumatic manifestations experienced by infected patients [3]. Since 2004, millions of cases of CHIKV infection have been reported in the Americas, Africa, Asia, Europe and Indian Ocean islands [4]. CHIKV outbreaks give rise to a grim economic burden on the affected regions, especially in the tropical and sub-tropical parts of the world, as the available treatment approaches, including fluid transfusion, bed rest and the use of antipyretics and analgesics can only alleviate the disease manifestation. In addition, vaccines against CHIKV have so far shown to be an intractable approach and there are no definite treatments against CHIKV infections [5]. Therefore, finding effective antiviral compounds against CHIKV is crucial.

In early studies, the methods and techniques used to examine antiviral agents included plaque reduction assay and MTT [3-(4, 5-dimethylthiazol-2-yl)-2, 5-diphenyltetrazoliumbromide] cell proliferation assay. Plaque reduction assay is still extensively practised as the gold standard for quantifying the lytic activity of viruses, which is observed in an infected confluent cell through macroscopic analysis of viral plaques prior to dye staining, with crystal violet, for example. The viral titres can be efficiently determined using this technique, as an end-point assay, although the method’s inadequacy regarding CPE onset and the kinetics of viral replication is markedly noted. Furthermore, infections with a diminished number of viruses and pH of the medium generate minute unclear plaques that are difficult to detect, or create no plaque in spite of virus replication [6].

MTT and MTS cell proliferation assays are enzyme-based assays that evaluate the activity of mitochondrial dehydrogenase in cells whereby mitochondrial NADH condenses MTT and also MTS to purple formazan. Basically, the colour concentration of formazan dye is associated with the number of vital cells [7]. However, these assays are time consuming in that they are labour intensive, requiring assessment by microscopic observation for quality control. Hence, an automated assay that monitors the biology of a cell in real-time is sought-after.

The xCELLigence real-time cell analysis (RTCA) system is an advanced technology, which allows real-time cell growth monitoring using a label-free cell-based assay that measures impedance variations in the culture media. This system has been applied in microbiological research [8], environmental toxicity [9] and cellular function [10]. Detailed and assorted aspects of cellular processes related to adhesion and morphology of cells, including cytotoxicity, cell proliferation, receptor-mediated signalling and migration can be analysed with RTCA [11]. In this system, to determine electronic impedance, cell culture wells are equipped with microelectrodes in the base, which attach to cultured cells through their sensors and record any changes. The microelectrodes are incorporated in special cell culture plates, E-Plates. In addition, the electrical impedance measured is based on cell status, termed CI [12]. The electrode impedance is exhibited and set down as the CI value to exemplify the differences in cell number, adhesion degree, cellular morphology and viability.

In our present study, we aim to investigate the efficacy of RTCA in the screening of antiviral drugs for CHIKV. Ribavirin, an approved *in vitro* inhibitor agent against CHIKV, was used in our investigation. Ribavirin has been shown to decrease CHIKV-induced arthritis and was found to be beneficial in resolving joint and soft tissue swelling [13, 14].

## Method

### Cell and virus

Vero cells, which are extracted from an African green monkey kidney-derived cell line CCL81, were obtained from the ATCC (Manassas, VA, USA). The adherent cell line was maintained in Eagle’s Minimum Essential Medium (EMEM; Gibco, Carlsbad, CA, USA) supplemented with 10 % foetal bovine serum (FBS; Gibco, Carlsbad, CA, USA). Cells were cultured in a humidified atmosphere with 5 % CO_2_ at 37 °C. The CHIKV strain used in this experiment was a clinical isolate from an outbreak in Johor in 2008, coded as MY/065/08/FN295485. It belongs to the ECSA genotype and has the A226V mutation in the E1 protein [15]. The CHIKV was then propagated on Vero cells, followed by the virus, using tissue culture infectious dose 50 (TCID_50_) methods [16]. Then, the virus stock was aliquoted and stored at −80 °C until needed. At the time of virus propagation and antiviral assays, the percentage of FBS in the cell culture media decreased to 2 %.

### Chemicals

Ribavirin (Sigma-Aldrich, St. Louis, MO, USA) and cisplatin (Sigma-Aldrich, St. Louis, MO, USA) were dissolved in dimethyl sulfoxide (DMSO, Sigma-Aldrich) to prepare 50 and 1 mg/ml stock solutions, respectively. The stock solutions were aliquoted and stored at −20 °C for future experiments.

### Real-time cell analysis system

The RTCA system comprises four main components: an electronic sensor analyser, a device station, a control unit and E-Plate 96. The software used in the study was RTCA Software 2.0 (Roche), which has the advantages of a user-friendly interface and electronic recording of the experimental details. The voltage for the RTCA analyser was between 100 and 240 V and the frequency was 50 to 60 Hz. The device station, which was placed inside the incubator, was able to switch any wells from E-plate 96 to the RTCA analyser for the impedance measurement. The CI value was a parameter that reflected the cell profile based on the impedance measurement. The CI value was zero in the absence or non-adherence of cells to the electrodes. In contrast, CI values increased gradually and consistently as cells attached to the electrodes. Additionally, the CI value measured the adherent degree of the cells. The degree of cell adhesion can be categorised into three degrees where a CI value of 1 to 4 is weak, 5 to 10 is considered moderate to strong and 10 to 15 denotes strongly adhered cells [17].

### Cell growth and proliferation assay using RTCA

The growth, proliferation and adhesion kinetics of Vero cells were determined using RTCA technology (ACEA Biosciences, San Diego, CA USA) as previously described with some minor modifications [18]. Briefly, 50 μl of EMEM supplemented with 10 % FBS (cell culture medium) was placed in each well of the E-plate 96 (gold-microelectrode array integrated E-plate; ACEA Biosciences, San Diego, CA USA). E-plate 96 was then connected to the system to obtain background impedance readings. This was to ensure that all wells of E-plate 96 and the connections were in good condition so as to avoid compromising the interpretation of the results. Serial dilutions of 2.0 × 10^4^, 1.8 × 10^4^ and 1.5 × 10^4^ cells in 50 μl were prepared, four replicates in each of the concentrations. These serial dilutions of cell suspensions were added to the wells containing 50 μl of culture medium. The E-plates were incubated at room temperature for 30 min in a laminar flow cabinet and then placed on the RTCA SP Station located in an incubator at 37 °C for continuous impedance recording. CI values measured by continuous impedance recordings every 2 minutes reflected the cell activities.

### Real-time monitoring of cytotoxicity assay of ribavirin and virus-induced cytopathogenicity using RTCA

RTCA was used to evaluate the *in vitro* cytotoxicity of ribavirin and also CHIKV-induced cytopathogenicity by profiling the adhesion, growth and proliferation kinetics of Vero cells in response to treatment. In brief, based on data from the cell growth and proliferation assay, 1.8 × 10^4^ cells were seeded in E-plate 96 followed by incubation at 37 °C with 5 % CO_2_. Proliferation, spreading and cell attachment kinetics were monitored every 2 minutes. When the cells reached the logarithmic growth phase, two-fold serial dilutions of ribavirin ranging from 200, 100, 50, 25 and 12.5 μg/ml were added to the wells of E-plate 96 in triplicate. The plate was then incubated at room temperature for 30 min and then placed on the RTCA SP Station for continuous impedance recording every 2 minutes.

As for virus titration assay, CHIKV stock was prepared in 10-fold dilutions at concentrations of 1 × 10^−1^, 1 × 10^−2^, 1 × 10^−3^, 1 × 10^−4^, 1 × 10^−5^ and 1 × 10^−6^ in EMEM supplemented with 2 % FBS. The plate was then incubated at 37 °C for 1 hour followed by washing with sterile PBS three times to remove the unabsorbed viruses. When the cells reached the logarithmic growth phase, the cell culture media in the wells were replaced by 100 μl of viral suspensions in triplicate. Then, 100 μl of EMEM supplemented with 2 % FBS was added to each well and the plate was placed on the RTCA SP Station at 37 °C with 5 % CO_2_ for continuous impedance recording every two minutes.

### Real-time cell growth profiling for antiviral assay using RTCA

RTCA was used to profile the adhesion, growth and proliferation kinetics for antiviral assay. Vero cells were seeded in E-plate 96 at a concentration determined from the cell growth and proliferation assay, and incubated at 37 °C. When the cells reached the logarithmic growth phase, the plate was detached from the RTCA SP Station. The remaining media were taken out from E-plate 96 and 100 μl virus suspension (MOI = 1) was added to each well. After 1 hour incubation at 37 °C viral inocula were replaced by 200 μl of increasing concentrations of the tested compounds, accordingly. Depending on the preliminary cytotoxicity data, ribavirin was prepared in serial dilutions of 50, 25, 12.5 and 6.25 μg/ml. For vehicle cell control, all the wells were replaced by adding the 200 μl of cell culture medium with 2 % FBS. E-Plate 96 was then incubated in the RTCA SP Station inside the incubator and the CI values were recorded every 2 minutes.

### Investigating acute drug responsiveness by RTCA

As a further study on the rapid detection of cytotoxicity by RTCA, Vero cells were treated with cisplatin as a compound with known potent cytotoxicity. The cells were prepared in EMEM supplemented with 10 % FBS and seeded in an E-Plate at a density of 1.8 × 10^4^ cells per well. The cisplatin was prepared in concentrations of 8, 6, 4, 2 and 0.5 μg/ml in EMEM supplemented with 2 % FBS. The plate was then incubated at room temperature for 30 min after adding the different concentrations of cisplatin in triplicate. Then, the plate was placed on the RTCA SP Station at 37 °C with 5 % CO_2_ for continuous impedance recording every 2 minutes.

### Cell morphology analysis

As a parallel comparison to both cytotoxicity and antiviral assay conducted by RTCA and MTS assay, the Vero cells were seeded in two different 96-well plates (Nunclon). The cell morphology was observed through inverted microscopy prior to MTS assay. The cells were visualised and their images were captured with a Zeiss Telaval 31 microscope after 72 h of incubation for both cytotoxicity and antiviral assays.

### Cytotoxicity assay of ribavirin and cisplatin using MTS

MTS assay as an approved cytotoxicity test was performed to determine the non-toxic concentrations of both ribavirin and cisplatin on Vero cells using an MTS (3-(4, 5-dimethylthiazol-2-yl)-5-(3-carboxymethoxyphenyl)-2-(4-sulfophenyl)-2H-tetrazolium) kit (Promega, USA) according to the manufacturer’s protocol. In short, the Vero cells were grown in 96-well plates and were treated with increasing concentrations of ribavirin and cisplatin in triplicate. The cytotoxicity assay was conducted for 48 and 72 h post-treatment, respectively. Subsequently, MTS solution was added to each well and incubated for 4 hours at 37 °C with 5 % CO_2_ followed by an absorbance reading at the 490 nm wavelength using Infinite 200 Pro-multiplate reader (Tecan, Männedorf, Switzerland).

### Antiviral assay using MTS

To identify the half maximal inhibitory concentration (IC_50_) of ribavirin against CHIKV-infected Vero cells, antiviral assay was conducted by MTS assay (Promega, USA) according to the manufacturer’s protocol. The Vero cells were grown in two different 96-well plates and were treated with different concentrations of ribavirin in triplicate after CHIKV inoculation of the cells. The plates were then incubated for 48 and 72 h at 37 °C with 5 % CO_2_ prior to MTS assay. Subsequently, MTS solution was added to the cells and incubated for 4 hours at 37 °C with 5 % CO_2_ followed by an absorbance reading at the 490 nm wavelength using the Infinite 200 Pro-multiplate reader (Tecan, Männedorf, Switzerland).

### Data analysis

The half maximal cytotoxic concentration (CC_50_) and inhibitory concentration (IC_50_) for the MTS assay was determined by Graph Pad Prism 5 (Graph Pad Software Inc., San Diego, CA, USA, 2005). A nonlinear regression was run to evaluate the association between the dose-dependent manner of the compound and antiviral activities of Vero cells. For RTCA data analysis, the normalised CI value was calculated for each sample well by normalising the CI value to a suitable time point prior to the treatment or infection. Fundamentally, the normalised CI value, CC_50_ and IC_50_ were automatically calculated by RTCA Software 2.0 (Roche). CI normalisation is essential to provide an accurate estimation of the percentage of cell adhesion, as it eliminates redundant data and secures the loading of the associated data.

## Results

### Monitoring of cell growth and proliferation

In this study, a cell proliferation analysis was performed using Vero cells to get the optimum cell number seeded in E-Plate 96 and to distinguish the optimal time point for viral infection prior to the antiviral assay. RTCA software was used to determine CI values through the measured impedance recordings.

**Fig. 1:**
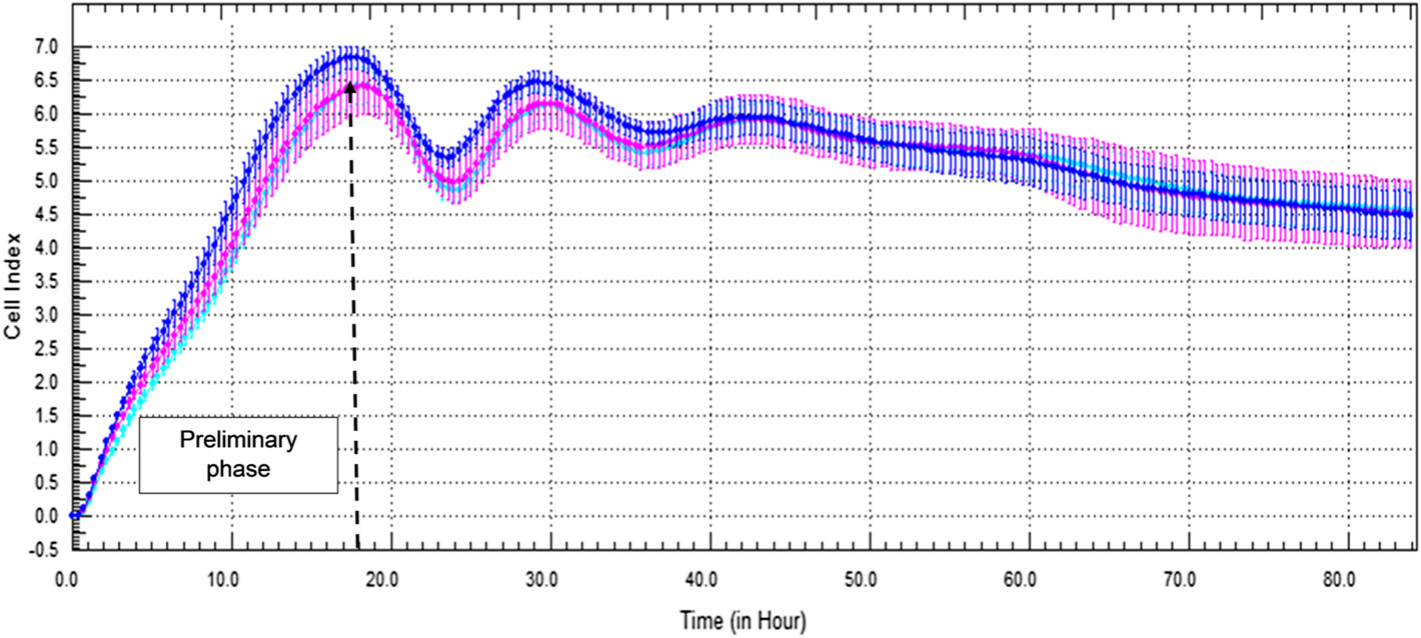
Proliferation Curve of Vero Cells. The cells that were seeded in E-Plate 96 and were constantly observed by measuring CI values to obtain the ideal cell number and to distinguish the suitable time point for virus infection prior to the antiviral assay are indicated. Cell features such as adhesion, spreading and proliferation were observed in intervals of 2 minutes. The dash line marks the initial phase of cell adhesion and spreading determined by the CI curve of 1.8 × 10^4^ cells at 18 h. Coloured curves represent the various numbers of cells seeded per well in E-Plate 96: Blue line: 2.0 × 10^4^ cells/well; Pink line: 1.8 × 10^4^ cells/well; Turquoise line: 1.5 × 10^4^ cells/well. Each data point signifies the average ± standard deviation and was analysed in triplicate

Three different cell densities of 2.0 × 10^4^, 1.8 × 10^4^ and 1.5 × 10^4^ cells were used in the experiment. As shown in Fig. 1, the preliminary phase of cell adhesion and proliferation was from 0 to 18 h after cell seeding, indicating the potential time point for the infection and treatment. The maximum CI value of 6.56 can be classified as a moderate to strong degree of cell adhesion (Fig. 1). The curves for all different densities of seeded Vero cells did not exhibit significant differences, therefore, as the median value of three different cell densities, 1.8 × 10^4^ was selected as the optimum number for cell seeding. The optimal time point for viral infection of 1.8 × 10^4^ cells was defined at 18 h after cell seeding.

### Cytotoxicity assay of ribavirin and cisplatin

**Fig. 2:**
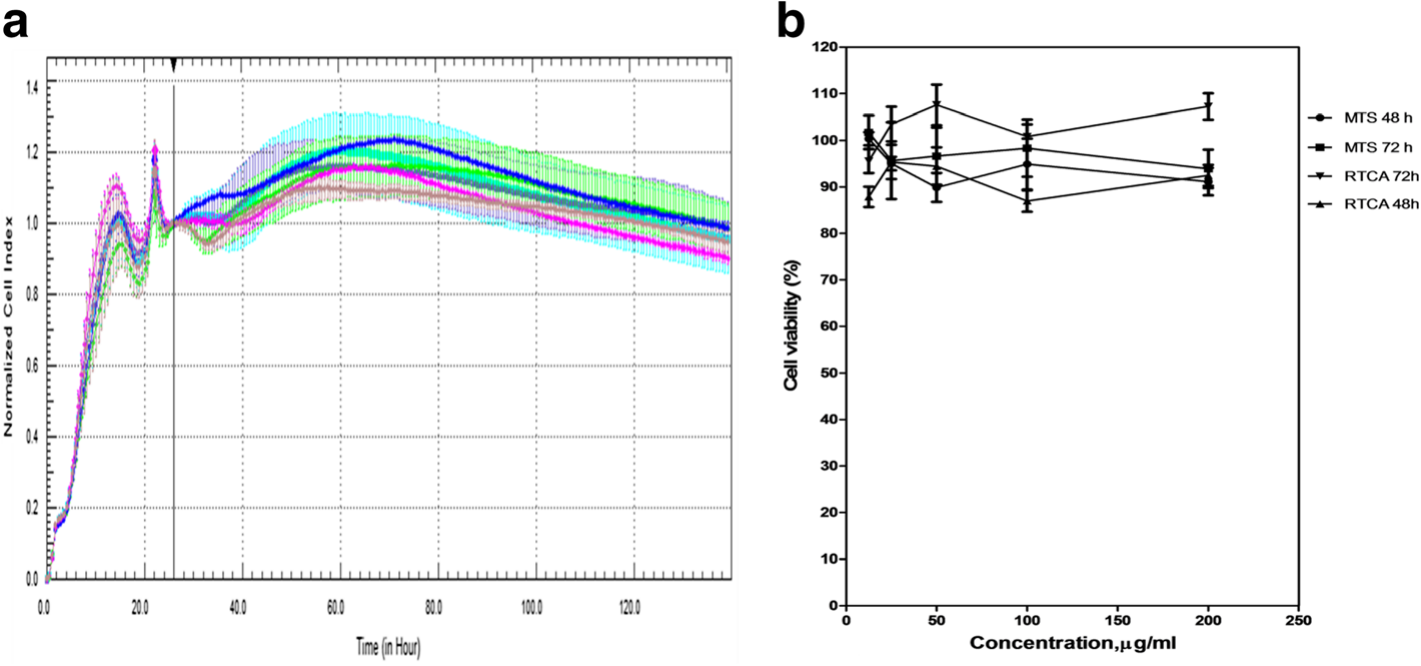
The Effect of the Intensified Concentrations of Ribavirin on Vero Cells Evaluated by both RTCA and MTS Assays. **a** The cell proliferation after 138 h of incubation with increasing concentrations of ribavirin. The black vertical line indicates the normalisation of the CI time point at 26 h subsequent to Vero cells being treated with ribavirin. Coloured curves represent the various serial dilutions of ribavirin. Each data point signifies the average ± standard deviation and was analysed in triplicate. Blue line: 200 μg/ml ribavirin; Pink line: 100 μg/ml ribavirin; Turquoise line: 50 μg/ml ribavirin; Purple line: 25 μg/ml ribavirin; Grey line: 12.5 μg/ml ribavirin; Green line: negative control (without ribavirin). Each data point signifies the average ± standard deviation and was analysed in triplicate. **b** In comparison to the RTCA assay, this figure indicates that the Vero cells were viable even when treated at the highest concentration of ribavirin on both days two and three, showing minimal cytotoxic effects of the compound on the cells via MTS assay

**Fig. 3:**
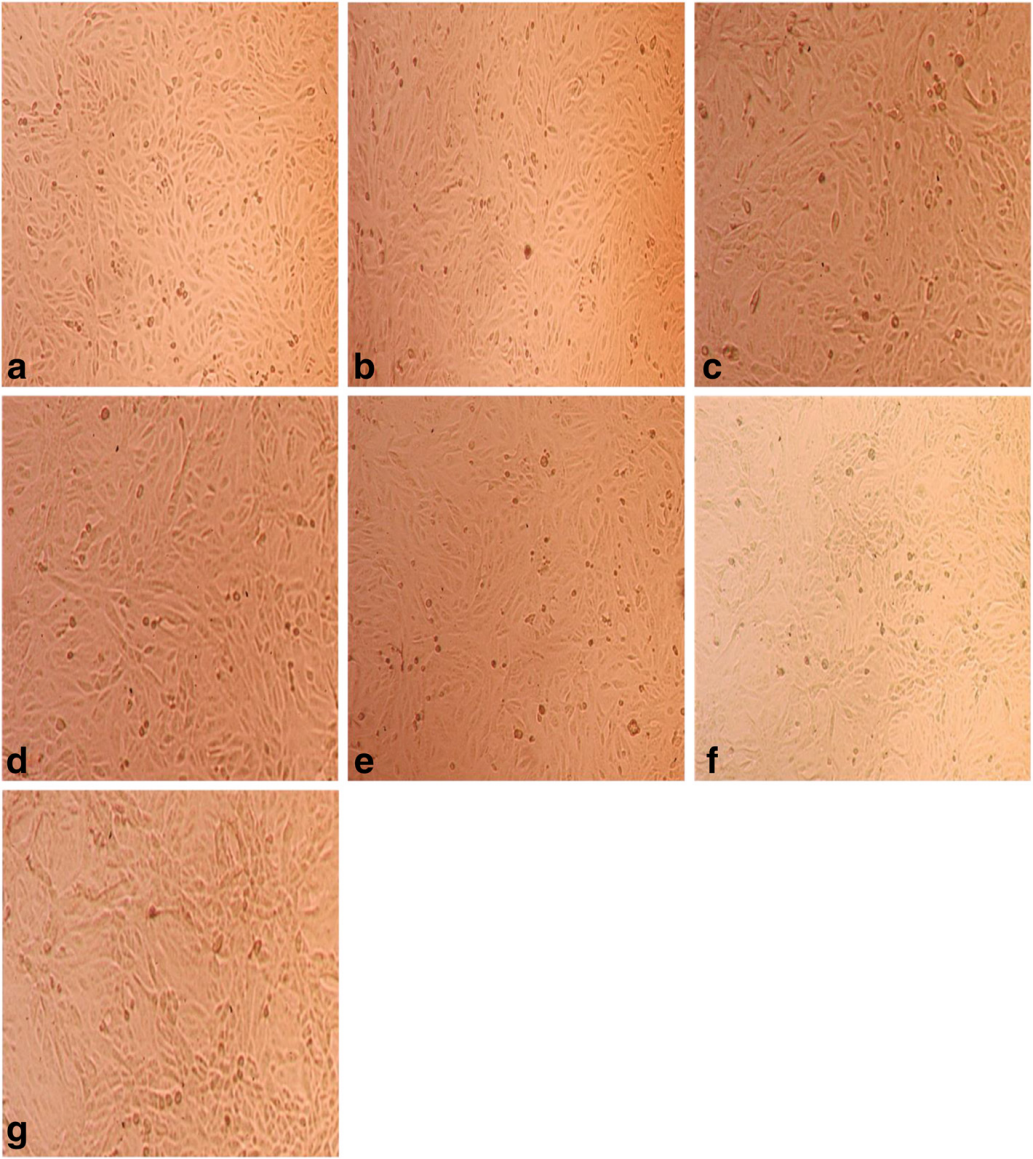
The Effect of the Intensified Concentrations of Ribavirin on Vero Cell Morphology. The cell morphology profile after 72 h of incubation after treatment with ribavirin. In parallel comparison to the cytotoxic effect on Vero cells, which was illustrated by RTCA, the cells seeded in a 96-well plate were observed via microscopy. Two-fold serially diluted ribavirin at concentrations of **a** 200 μg/ml, **b** 100 μg/ml, **c** 50 μg/ml, **d** 25 μg/ml, **e** 12.5 μg/ml and **f** 6.25 μg/ml were used to treat the Vero cells. As a control, **g**, three wells of non-treated cells were monitored

Cytotoxicity assay was performed to identify the CC_50_ of the compounds. The cytotoxicity of ribavirin and cisplatin was analysed by both RTCA and MTS methods. It was found that there was no significant cytotoxicity for all tested concentrations of ribavirin in Vero cells. The optimal treatment time point for both compounds was at approximately 19 h. The CI value was normalised to the suitable time point after treatment, which was 26 h, so as to eliminate the redundant data. The results showed that more than 90 % of the cells treated with 200 μg/ml of ribavirin were viable (Fig. 2). The curves in Fig. 2a illustrated by RTCA show that the ribavirin does not restrict the impedance measurement even at the highest concentration, which indicates that 200 μg/ml of ribavirin does not show toxicity in Vero cells. We have also shown that ribavirin treated-Vero cells do not display significant changes in cellular morphology (Fig. 3).

**Fig. 4:**
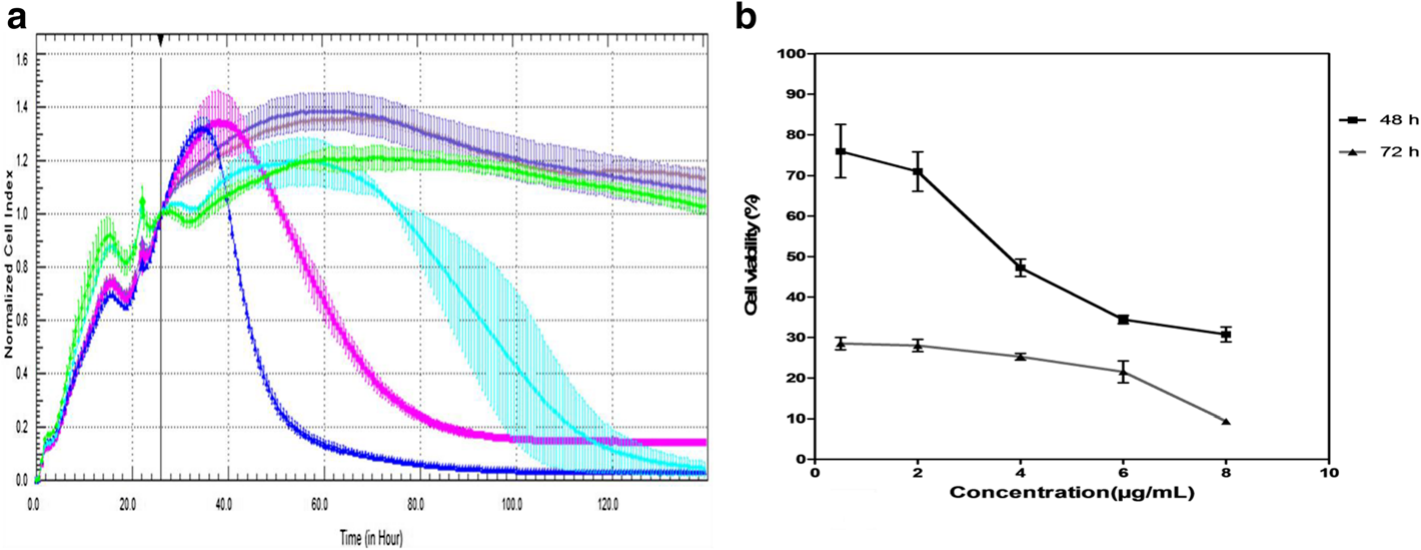
The Effect of the Intensified Concentrations of Drug-Induced Cytotoxicity on Vero Cells Evaluated by both RTCA and MTS Assays. **a** Vero cell profile after 138 h of incubation with increasing concentrations of cisplatin. The black vertical line indicates the normalisation of the CI time point at 26 h subsequent to Vero cells being treated with cisplatin. Coloured curves represent the various concentrations of cisplatin. Each data point signifies the average ± standard deviation and was analysed in triplicate. Blue line: 8 μg/ml cisplatin; Pink line: 6 μg/ml cisplatin; Turquoise line: 4 μg/ml cisplatin; Purple line: 2 μg/ml cisplatin; Grey line: 0.5 μg/ml cisplatin; Green line: negative control (without cisplatin). Each data point signifies the average ± standard deviation and was analysed in triplicate. **b** Illustration of the curve of cisplatin effects on Vero cells at 48 and 72 h via MTS assay

The results for cytotoxicity of cisplatin in Vero cells are illustrated in Fig. 4. It has been shown that approximately 15 h post-treatment, the CI value of Vero cells treated with 8 μg/ml of cisplatin start to decline, followed by cells treated with 6 μg/ml of cisplatin at 21 h post-treatment (Fig. 4a). The CC_50_ obtained from RTCA was 5.620 μg/ml for both days two and three post-treatment, respectively. Nevertheless, the CC_50_ values resulting from MTS assay were 3.409 and 0.033 μg/ml for days two and three post-treatment, respectively (Fig. 4b).

### Antiviral assay of ribavirin against CHIKV

**Fig. 5:**
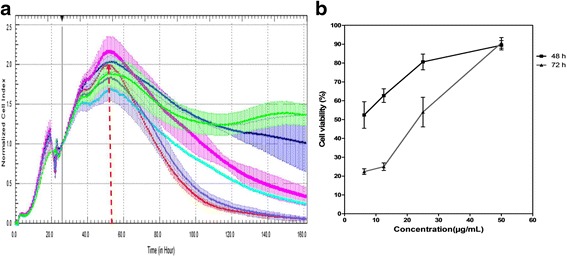
The Antiviral Effect on Vero Cells Infected with CHIKV Evaluated by both RTCA and MTS Assays. **a** Measurement of cell impedance every 2 minutes by RTCA monitored the antiviral effect of the various concentrations of ribavirin on the proliferation, spreading and adhesion of cells. The black vertical line indicates the normalisation of the CI time point at 26 h subsequent to treatment of infected Vero cells with ribavirin. The red dash line indicates the onset of declined curved due to infectivity of CHIKV and antiviral activity on Vero cells. Coloured curves represent the Vero cells, viral infected Vero cells and treated viral infected Vero cells with different concentrations of ribavirin. Blue line: 50 μg/ml ribavirin; Pink line: 25 μg/ml ribavirin; Turquoise line: 12.5 μg/ml ribavirin; Purple line: 6.25 μg/ml ribavirin; Red line: positive control (CHIKV infection); Green line: negative control (without CHIKV infection). Each data point signifies the average ± standard deviation and was analysed in triplicate. **b** The curve shows the antiviral activity effects via MTS assays, which can only be obtained at 48 and 72 h prior to ribavirin treatment of CHIKV-infected Vero cells

**Fig. 6:**
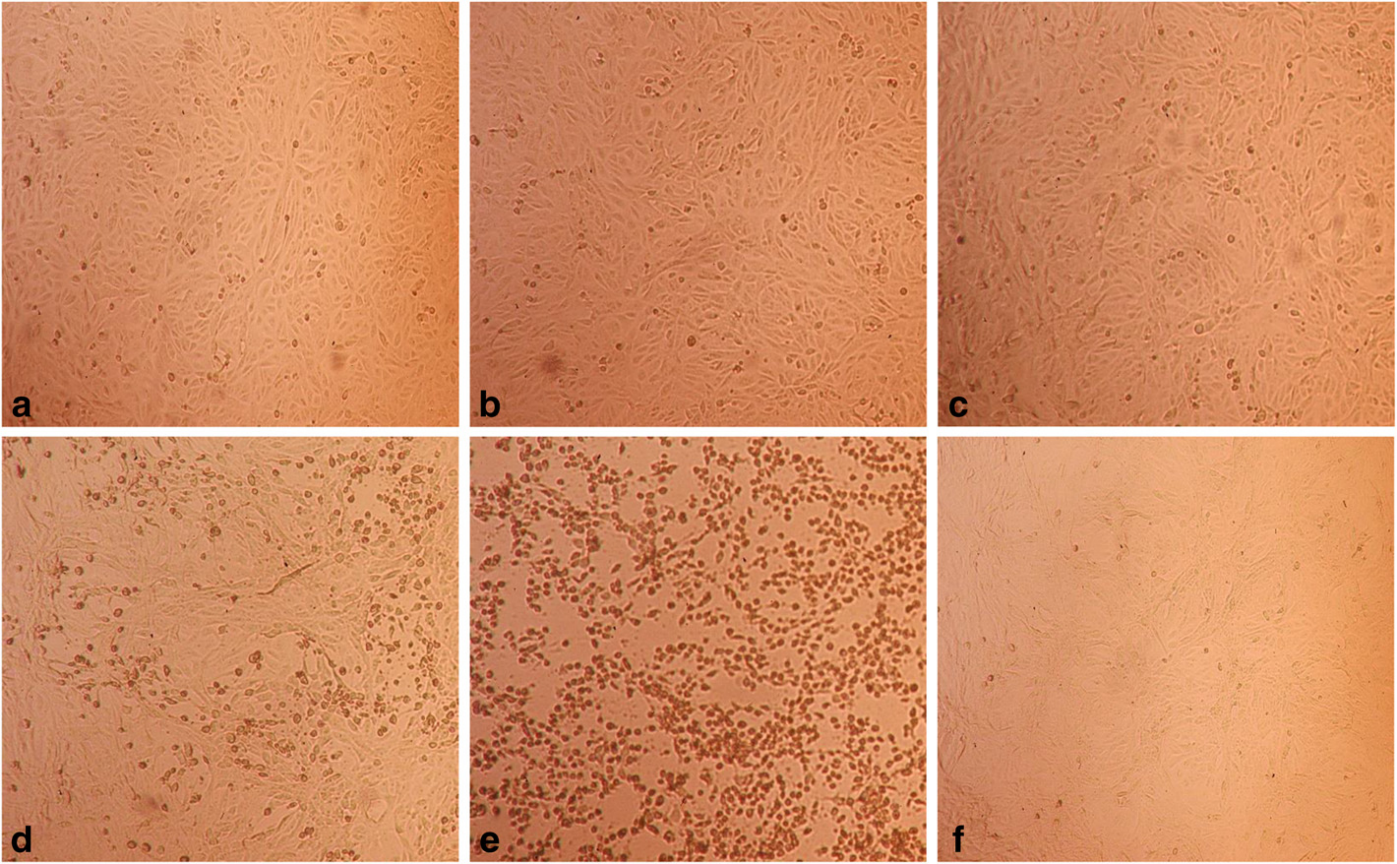
The Antiviral Effect on Vero Cell Morphology. Cell morphology profile after 72 h post infection and treatment. In parallel comparison to the antiviral effect on Vero cells, which was indicated by RTCA, the cells were seeded in a 96-well plate and observed via microscopy. CHIKV-infected cells were treated with ribavirin at concentrations of **a** 50 μg/ml, **b** 25 μg/ml, **c** 12.5 μg/ml, and **d** 6.25 μg/ml. As a virus control, three wells of non-treated CHIKV-infected cells, (**e**), were used. As vehicle control, three wells of non-infected Vero cells, (**f**), were monitored

Eighteen hours after cell seeding, Vero cells were infected with CHIKV followed by treatment with ribavirin. The results from RTCA showed that the CI values of CHIKV-infected Vero cells started to decrease 34 h post-infection, while vehicle controls and treated infected-Vero cells did not show any significant changes (Fig. 5a). The results also indicated compliance with the dose-dependent manner for ribavirin treatment, which was verified by the CI values of Vero cells-infected with CHIKV. The data from MTS assay demonstrated that there was no significant change that could be observed in all concentrations of ribavirin-treated cells except for in the lowest concentration of ribavirin (Fig. 5b). This result can also be verified by the cell morphology profiles in Fig. 6. The MTS result exemplified that the IC_50_ for ribavirin at 48 and 72 h were 6.12 and 20.34 μg/ml, respectively. Meanwhile, the IC_50_ interpreted by RTCA was 14.78 μg/ml at both 48 and 72 h.

### Viral titration assay

**Fig. 7:**
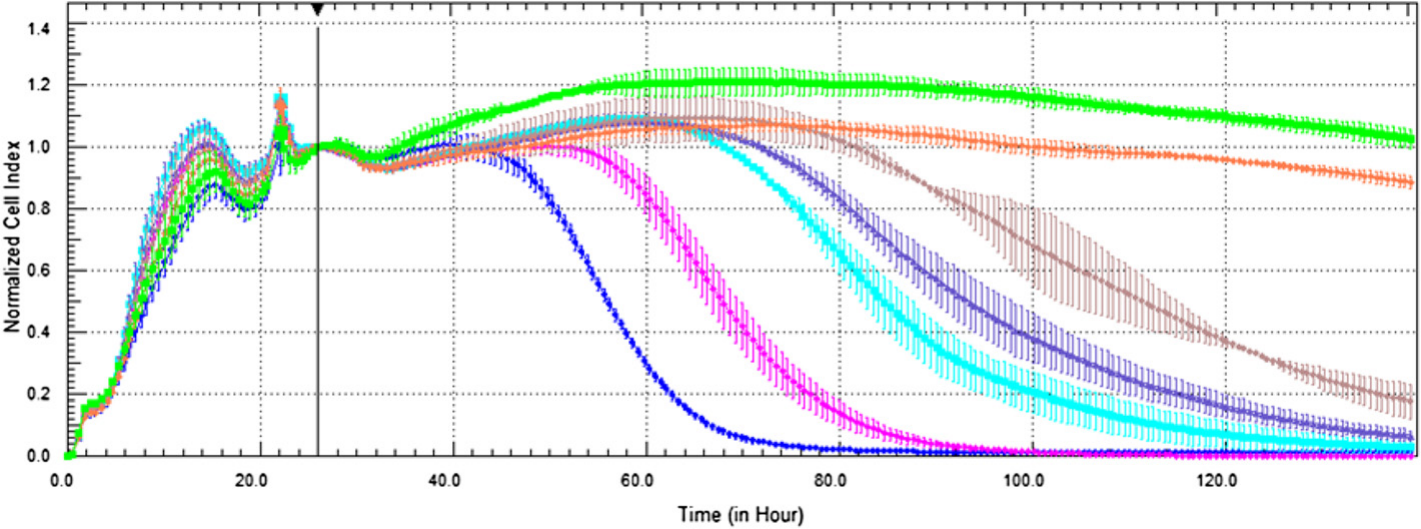
The Viral Titration of Vero Cell Infected with CHIKV Evaluated by RTCA. Measuring cell impedance every 2 minutes by RTCA monitored the viral dose-dependency effect of various dilutions of CHIKV inoculum on the proliferation, spreading and adhesion of cells. The black vertical line indicates the normalisation of the CI time point at 26 h subsequent to CHIKV infection of the Vero cell line. Coloured curves represent the Vero cells infected with different concentrations of CHIKV. Blue line: 10^−1^ CHIKV; Pink line: 10^−2^ CHIKV; Turquoise line: 10^−3^ CHIKV; Purple line: 10^−4^ CHIKV; Grey line: 10^−5^ CHIKV and Orange line: 10^−6^ CHIKV; Green line: negative control (without CHIKV infection). Each data point signifies the average ± standard deviation and was analysed in triplicate

Viral titration assay was conducted to verify and optimise the viral dose-dependency of CHIKV on RTCA technology. As shown in Fig. 7, the graph curves started to decrease at 18 h after infection for the highest virus dilution, which was 1 × 10^−1^,and decreased gradually as the dilutions increased, exhibiting a dose-dependent manner for CHIKV infection in Vero cells. The decrease in CI value indicated cell death as a consequence of CHIKV replication, while in contrast, vehicle control and Vero cells infected with 1 × 10^−6^ dilution of CHIKV did not show any significant change in CI value (Fig. 7). In parallel to this experiment, TCID_50_ of CHIKV was performed in a 96-well plate for the same series of virus concentrations. The TCID_50_ obtained was 10^3.5^ EID_50_/ml for both RTCA and conventional end-point assay.

## Discussion

Nowadays, researchers are adopting advanced technology, such as label-free detection methodologies for cell-based experiments [19]. Cellular activities, including growth inhibition, shape change, spreading, migration and adhered cell degree, can be effectively determined by RTCA technology. Moreover, RTCA is favoured for the efficacy and safety of the experiments [10]. This technology is reasonably consistent and sufficiently dynamic for wide use in medium-throughput screening.

Therefore, in the present study, we aimed to prove the ability of RTCA to detect optimal culture conditions and to examine the reproducibility of cytotoxicity and antiviral assays. Based on the cell growth and proliferation profile measured by RTCA, the curves of initial adhesion of Vero cells were characterised by the sharp rise in CI value over the 18 h post-cell seeding and followed by a proliferation period that started to decrease at approximately 48 h post-cell seeding. The decrease in CI value indicated that the cells were not healthy and cell death was possible. Based on our findings through classic end-point methods, such as MTT and MTS assays for the CHIKV antiviral assay, which was 72 h post-infection, we set up the same end point for the reading of our developed RTCA method. Information on behaviour, propagation and fitness of cells through CI value curves was beneficial and enhanced the experimental design compared to conventional *in vitro* methods, such as MTT and MTS assays whereby the treatments were commonly introduced at a certain time point, technically 24 h after cell seeding in the plates [13].

As reported by Briolant et al. ribavirin is known as an effective antiviral agent against CHIKV *in vitro* replication with minimal cytotoxic effects on Vero cells. The results obtained from both methods used in this study showed no cytotoxicity for ribavirin on Vero cells up to 200 μg/ml of the compound. Cisplatin is a chemotherapy drug widely used to treat different types of cancers such as sarcoma, lung cancer and ovarian cancer. But it can become toxic at certain concentrations for different cells. Therefore, we used cisplatin as a control with known cytotoxicity. Our cytotoxicity evaluation of cisplatin through the RTCA method showed the efficacy and sensitivity of RTCA in detecting cytotoxic effects in a real-time monitoring frame and a well-defined dose-dependent manner.

In this study, we observed different times of onset of CPE for both RTCA and MTS assay as a conventional end-point assay. The time point for onset of CPE for antiviral assay evaluated by RTCA was at approximately 14 h earlier compared to the MTS. The curve generated by the RTCA software showed a decline at 34 h post-infection, while for the MTS assay, at least 48 h passed before significant changes could be identified through the absorbance reading. The IC_50_ of the conventional assay exhibited vast differences between both days of treatment compared to the IC_50_ computed by RTCA, which showed a consistent and precise result through real-time monitoring. The graph from the MTS assay did not show resilient significant changes in ribavirin treatment at 48 and 72 h. However, we observed a dose-dependent manner curve in RTCA analysis after ribavirin treatment for CHIKV-infected Vero cells, as the CI value started to decrease at approximately 34 h post infection and treatment. This finding indicated that RTCA technology was capable of detecting real-time changes in quantification of virus-induced CPE and the behaviour of the cellular mechanism when exposed to a drug or compound, which cannot be observed through the conventional methods.

Cell quality control and stability are major concerns due to the amplified dependence on cells in drug discovery [20, 21]. In traditional methods, phenotypic analysis and viability are examined by microscopy and dye staining techniques, which, in addition to being time-consuming, are mainly carried out at a single time point. Kirstein et al. [20] have shown similar proliferation kinetic profiles for various cell types, which explains a quantitative account of their cellular behaviour. In comparison to conventional cell-based assays, RTCA is a practical method whereby all the information regarding cell activities throughout the incubation period is automatically recorded and monitoring is continuous.

As a diagnostic tool, this information can be effectively used for cellular quality control, which could be measured based on the RTCA technology. To encourage more investigators to use RTCA in primary toxicity screening, emphasising the close correlation between cellular impedance measurements with numerous compounds is considered to be vital. However, the limited usage of RTCA assay, which works best with adhered cells and comes at a great expense in comparison with traditional facilities, is a restricting factor for RTCA.

## Conclusion

In conclusion, regardless of restrictions, RTCA technology seems to be a prevailing and trustworthy tool in research innovation due to the realistic throughput, the opportunity to carry out quality control and technical optimisation, and the likelihood of analysing real-time cell reactions to antiviral agents for the development of pharmaceutical products in the early stages [22]. This system can produce predictive facts at the hit-to-lead phase and as a result, diminish the destruction rate due to the safety and efficacy associated with substantial savings for the advanced stages of antiviral drug development [23]. In addition, unlike the conventional end-point assays, RTCA provides time-based biological information at the interface between cells and toxicants.

## Abbreviations

RTCA: Real-time cell analyser
CHIKV: Chikungunya virus
CPE: Cytopathic effects
DMSO: Dimethyl sulfoxide
CI: Cell Index
MNTD: Maximum non-toxic dose
TCID_50_: Tissue culture infectious dose
IC_50_: Inhibitory concentration
CC_50_: Cytotoxicity concentration.
